# Cross-talk between the cytokinin, auxin, and gibberellin regulatory networks in determining parthenocarpy in cucumber

**DOI:** 10.3389/fgene.2022.957360

**Published:** 2022-08-26

**Authors:** Neha Kumari Mandal, Khushboo Kumari, Aditi Kundu, Ajay Arora, Prolay K. Bhowmick, Mir Asif Iquebal, Sarika Jaiswal, Tusar Kanti Behera, A. D. Munshi, Shyam S. Dey

**Affiliations:** ^1^ Division of Vegetable Science, ICAR-Indian Agricultural Research Institute, New Delhi, India; ^2^ Division of Agricultural Chemicals, ICAR-Indian Agricultural Research Institute, New Delhi, India; ^3^ Division of Plant Physiology, ICAR-Indian Agricultural Research Institute, New Delhi, India; ^4^ Division of Genetics, ICAR-Indian Agricultural Statistics Research Institute, New Delhi, India; ^5^ Centre for Agricultural Bioinformatics, ICAR-Indian Agricultural Statistics Research Institute, New Delhi, India; ^6^ ICAR-Indian Institute of Vegetable Research, Varanasi, India

**Keywords:** cucumber, parthenocarpy, slicing and pickling cucumber, cytokinin, gene expression, endogenous level, exogenous spray

## Abstract

Cucumber is a model plant for studying parthenocarpy with abundant slicing- and pickling-type germplasm. This study was undertaken to understand the role of the important cytokines (CKs), auxin (AUX) and gibberellin (GA) biosynthesis and degradation genes for the induction of parthenocarpy in slicing and pickling germplasm. Two genotypes of gynoecious parthenocarpic cucumber, PPC-6 and DG-8, along with an MABC-derived gynoecious non-parthenocarpic line, IMPU-1, were evaluated in this study. The slicing and pickling cucumber genotypes PPC-6 and DG-8 were strongly parthenocarpic in nature and set fruit normally without pollination. Endogenous auxin and gibberellin were significantly higher in parthenocarpic than non-parthenocarpic genotypes, whereas the concentration of cytokinins varied among the genotypes at different developmental stages. However, the exogenous application of Zeatin and IAA + Zeatin was effective in inducing parthenocarpic fruit in IMPU-1. Expression analysis with important CK, AUX, and GA biosynthesis-related genes was conducted in IMPU-1, PPC-6, and DG-8. The expression of the CK synthase, *IPT, IPT3*, *PaO, LOG1, LOG2, CYP735A1,* and *CYP735A2* was up-regulated in the parthenocarpic genotypes. Among the transcription factor response regulators (RRs), positive regulation of *CSRR8/9b, CSRR8/9d, CSRR8/9e*, and *CSRR16/17* and negative feedback of the CK signalling genes, such as *CsRR3/4a, CsRR3/4b, CsRR8/9a*, and *CsRR8/9c*, were recorded in the parthenocarpic lines. Homeostasis between cytokinin biosynthesis and degradation genes such as CK oxidases (*CKXs*) and CK dehydrogenase resulted in a non-significant difference in the endogenous CK concentration in the parthenocarpic and non-parthenocarpic genotypes. In addition, up-regulation of the key auxin-inducing proteins and GA biosynthesis genes indicated their crucial role in the parthenocarpic fruit set of cucumber. This study establishes the critical role of the CKs, AUX, and GA regulatory networks and their cross-talk in determining parthenocarpy in slicing and pickling cucumber genotypes.

## Introduction

Successful pollination and fertilization in flowering plants are integral to fruit setting and development. However, a few plant species exhibit a typical mechanism for successful fruit setting and development without fertilization—termed parthenocarpy (Zinn et al., 2010; Knapp et al., 2017). Elongation of the pollen tubes and development of seeds result in the release of several phytohormones which facilitate the fruit set and enlargement of ovaries (Crane, 1964; Ozga et al., 2003). Cucumber (*Cucumis sativus* L.) is a model plant species for genetic and genomic study (Li et al., 2014); some of the genotypes of this important vegetable crop have the ability of parthenocarpic fruit development. Cucumber is the fourth most important vegetable crop and is cultivated in more than 150 counties around the world. It has emerged as the top crop for protected cultivation because of its ability to set fruit without pollination. The productivity of parthenocarpic cucumber cultivars is much higher than that of non-parthenocarpic monoecious genotypes which require successful cross-pollination for fertilization and fruit development. Cucumber has emerged as a model organism for understanding parthenocarpy because of its abundant parthenocarpic germplasm. Fruit set without pollination in a gynoecious genotype with only female flowers has enabled a manifold increase in cucumber productivity, giving it high potential as an economic crop for protected cultivation (Sharif et al., 2022).

Parthenocarpy in cucumber is a complex phenomenon controlled by a series of genetic mechanisms and the endogenous concentration of phytohormones (Gou et al., 2022; Sharif et al., 2022). The interest in parthenocarpic traits in horticultural crops is increasing because of their importance in improving fruit quality, tolerance to biotic and abiotic stresses, and reduced fruit drop. The development of seedless fruit results in greater flesh content as seeds and seed cavities are replaced by edible pulp or expended mesocarp (Liu et al., 2018). Endogenous levels of important plant hormones in the ovaries are reported to be closely associated with the parthenocarpic development of fruit pulp (Li et al., 2014; Su et al., 2021). Cross-talk between auxins (AUX), cytokinin (CKs) and gibberellins (GAs) in fruit set and parthenocarpic fruit development have been reviewed by Kumar et al. (2014) and Sharif et al. (2022) in important horticultural crops.

CKs play a major role in fruit set and their further development. It is observed that many plants accumulate high concentrations of endogenous CKs during the development of fruit; its exogenous application promotes parthenocarpic fruit development (Lu et al., 2016; Qian et al., 2018; Chai et al., 2019). CK stimulates cell division during fruit development and promotes cell proliferation in the ovarian tissues. CK also increases initial fruit set and delays fruit abscission in the case of parthenocarpic fruit. In the absence of pollination and fertilization, where the process of fruit set is about to fail, high concentrations of CKs can promote the quick proliferation of ovarian tissue and the retention of ovaries to develop into fully grown fruits (Kim et al., 1992). Homeostasis between CK synthesis and catabolism determines spatial and temporal biosynthesis (Frébort et al., 2011). CKs are reported to promote parthenocarpy in many fruits and vegetables such as tomato, cucumber, watermelon, eggplant, grape, and fig. In the case of tomato, concentrations of cytokinin ribosides and isopentenyladenine, and transcript levels of CK biosynthetic genes such as *SlPT3*, *SlPT4,* and *SlLOG6* were high during anthesis (Ren et al., 2011; Matsuo et al., 2012). Concentrations of trans-zeatin and transcript levels of *SlPT1*, *SlPT1*, *SlLOG2,* etc. were increased after anthesis (Matsuo et al., 2012). Indole-3-acetic acid (IAA) is reported as having the potential of inducing parthenocarpy in important vegetable crops like tomato, cucumber and zucchini (Martinelli et al., 2009; Pomares-Viciana et al., 2017). In addition, a single gene such as a transcriptional factor or a receptor in phytohormone signalling pathways can also control parthenocarpy (Martí et al., 2007; Serrani et al., 2010; Fuentes et al., 2012). The auxin response factor (ARF) and AUX/IAA are two important auxin-responsive gene families reported to be related to parthenocarpic fruit development in *Arabidopsis* and tomato (Kumar et al., 2014). Among the different AUX biosynthesis pathways, the role of *Trp-IPyA* (tryptophan-indole-3-pyruvic acid) has been established in the development of parthenocarpic fruits. The role of the *YUCCA10*, *PavYUCCA10*, *SlTAR1*, *ToFZY2*, *ToFZY3,* and *PARENTAL ADVICE-1 (PAD-1)* genes in the parthenocarpic fruit development of loquat, tomato and eggplants have been reviewed in detail by Sharif et al. (2022). The cultivation of GA signalling in ovules and valves was reported to be because of fertilization-triggered AUX signalling in *Arabidopsis* (Dorcey et al., 2009). Interaction among AUX and GA signalling pathways is also reported to be essential for fruit set and development (Srivastava and Handa, 2005; de; Jong et al., 2011; Carrera et al., 2012; Ruan et al., 2012). Auxin biosynthesis genes encoding proteins such as *YUCCA5*, *YUCCA11*, and tryptophan aminotransferase-related 1 and GA biosynthesis genes encoding enzymes such as *GA 20-oxidase3* and *GA 3-oxidase3, 4, 5*, and *6* are reported to play important an role in fruit set and development in strawberry (Kang et al., 2013).

The mechanisms of parthenocarpy in cucumber have been the subject of a number of studies (Fu et al., 2008; Li et al., 2014; Su et al., 2021; Gou et al., 2022). In cucumber, the nature of parthenocarpy is classified as facultative with the ability of fruit set without pollination. However, the parthenocarpic genotypes can successfully produce seeds when pollinated to effect fertilization. The inheritance of parthenocarpy in cucumber is reported to be governed by single dominant genes to complex polygenes (Pike and Peterson, 1969; Ponti and Garretsen, 1976; Kim et al., 1992). Most of the recent studies indicated that a large number of QTLs are associated with parthenocarpic fruit development of in cucumber. In the case of European greenhouse slicing cucumber, parthenocarpy was reported to be controlled by seven QTLs, including one major QTL on chromosome 2 (Wu et al., 2015). In North American pickling-type cucumber 2A, seven QTLs were detected for parthenocarpy and one QTL each on chromosomes 5 and 7 (parth5.1 and parth7.1), and two on chromosome 6 (parth6.1 and parth6.2) (Lietzow et al., 2016). Recently, four novel QTLs associated with parthenocarpy were detected in South China ecotype cucumber (Niu et al., 2020). These studies depict the complex genetic mechanisms associated with parthenocarpy in cucumber. Different ecotypes in cucumber, including slicing and pickling types, also had a different genetic architecture determining parthenocarpy.

There is a consensus based on earlier reports that parthenocarpy is a complex trait. Different genomic regions and QTLs were identified as determining parthenocarpy in slicing and pickling/processing-type cucumbers (Wu et al., 2015; Lietzow et al., 2016; Niu et al., 2020). However, there have been no reports regarding the comparative analysis of parthenocarpy in two different groups of cucumbers: slicing and pickling. These two groups have a different evolutionary lineage and, therefore, understanding the role of important phytohormones and genetic mechanisms of parthenocarpy need to be studied further. The present study involved two parthenocarpic gynoecious lines and one non-parthenocarpic gynoecious line for better insight into parthenocarpy in cucumber genotypes. The study aimed to determine the role of the important phytohormones’ CK, AUX and GA biosynthesis and degradation-related genes in determining parthenocarpy in slicing- and pickling-type cucumber genotypes.

## Materials and methods

### Plant materials and growing conditions

The present experiment was undertaken using two gynoecious parthenocarpic genotypes and one gynoecious non-parthenocarpic genotype. The genotype Pusa Parthenocarpic Cucumber-6 (PPC-6) is a commercially cultivated slicing type suitable for cultivation under protected conditions. Pusa Pickling Cucumber-8 (DG-8) was the second gynoecious parthenocarpic genotype which is a speciality genotype suitable for pickling and cultivation under protected conditions. The non-parthenocarpic genotype, IMPU-1, was developed through introgression of *F* locus-determining gynoecy into a commercially cultivated elite monoecious genotype, Pusa Uday, through marker assisted breeding (MABC) (Behera et al., 2022). All the genotypes under investigation were gynoecious in nature, thus enabling a precise analysis of important phytohormones and determining the role of different CK, AUX and GA biosynthesis and degradation genes in inducing parthenocarpy in different groups of cucumber. All the genotypes were grown under protected conditions with standard recommended practice for the protected cultivation of cucumber.

### Exogenous application of auxin, cytokinin, and gibberellin for parthenocarpic fruit development

The parthenocarpic and non-parthenocarpic genotypes PPC-6, DG-8, and IMPU-1 were grown in three replications with five plants per genotype for recording parthenocarpic fruit development under control with no exogenous application of phytohormones. Fruit set was recorded for the fifth node onwards for 15 fruits in each plant. An average of five plants in each replication was taken for analysis. In an unpollinated condition, the length of the fruit was measured from ten fruits in each plant. The exogenous application of IAA, Zeatin and GA_3_ was done in the genotype IMPU-1 in 15 fruits in each plant to reach 20 nodes, starting from the sixth node. For exogenous application, seven treatments—IAA, GA_3_, Zeatin, IAA + GA_3_, IAA + Zeatin, Zeatin + GA_3_, and IAA + GA_3_+Zeatin—were applied. Exogenous application was done on the day of flower anthesis before opening the flowers in the early morning between 6:00 and 7:00 a.m. Flower buds sprayed with different combinations of phytohormone spray were covered with butter paper to avoid any chance of pollination. Three growth hormones were initially applied alone at four different doses—25, 50, 100, and 150 mg/lit—before the start of the experiment in the genotype IMPU-1; it was observed that a concentration of 100 mg/lit exhibited better response in parthenocarpic fruit set. Therefore, each of the phytohormones were sprayed with a concentration of 100 mg/lit to observe parthenocarpic fruit development. Fruit set was observed to ten days after anthesis and application of the phytohormones. As there was sequential flowering in all the lines, three to five fruits were sprayed in a single day in each plant and 15 female buds in each plant were taken for data recording. The experiment was conducted in three replications with five plants in each replication. The fruits in each plant were removed after recording of the data on 10 DAA to allow other fruits in the plant to develop. Average data of each replication analysed using STAR software (http://bbi.irri.org › products). Tukey’s honest significant difference (HSD) at *p* = 0.05 was used to determine the test of significance.

### Estimation of endogenous IAA content

The frozen fruit samples at different developmental stages (5 g) were powdered in liquid nitrogen. The protocol described by Kim et al. (2006) was followed to extract IAA from the fruit samples. These were ground with liquid nitrogen and extracted with 100% methanol (2.5 ml g^−1^ fresh weight). The prepared extract was centrifuged at 16,000 g for 10 min. at 4°C. A vacuum concentrator was used to prepare a concentrate of the resulting supernatant. The conditions for HPLC were optimised and used for IAA quantification (Sharma et al., 2018) with an injection volume of 20 μL for each sample. A standard IAA sample was obtained from Sigma-Aldrich and final concentration was represented as μg IAA g^−1^ FW.

### Estimation of trans-zeatin and dihydrozeatin

Trans-Zeatin and dihydrozeatin were extracted using a modified method suggested by Arteca et al. (1980). Fruit samples (10 g each) were submerged in 80% (v/v) methanol in water and extracted using a ultrasonicator (VCX-750, Sonics, Sonics and Materials Inc., Newtown, United States) at 50 kHz for 20 min. Aqueous methanolic extracts were centrifuged at 25000 g for 20 min separately and the supernatant was concentrated in a vacuum using a rotary evaporator (Heidolph, Germany) at 40°C. The remaining solution was partitioned with 10 ml of acetonitrile (0.1% TFA). Acetonitrile soluble fraction was subjected to UPLC-QTOF-ESIMS analysis in a Acquity Ultra Performance Liquid Chromatograph, coupled to a Quadrupole-Time of Flight mass spectrometer (QToF-MS, Synapt G2 HDMS, Waters Corporation, Manchester, United Kingdom). Reference standards of trans-zeatin [(purity 99.5%)] and dihydrozeatin (purity 99%) were used to prepare respective calibration curves for estimation. The QToF-ESI-MS was operated with electrospray ionization (ESI) at a nominal mass resolution of 20,000 and controlled by MassLynx 4.1 software. The data acquisition was done with the MSE function in continuum mode in the range of m/z 50–1000. The MSE mode provides full-scan MS data (low energy, 4 V) and MS/MS data (high energy, 10–60 V ramping) simultaneously. The source parameters were set as follows: capillary 6 kV, sampling cone 30 V, extraction cone 5 V, source temperature 100°C, desolvation temperature 500°C, desolvation gas flow 1000 L h^-1^ and cone gas flow 50 L h^-1^. For mass spectrometer calibration, 0.1 mM sodium formate was used. The lock spray, the reference mass leucine enkephalin (m/z 556.2771 in positive and m/z 554.2670 in negative polarity) at 1 µg ml^-1^ concentration was used for mass correction with a flow rate of 20 µL min^-1^ in every ten desolvation gas flow 1000 L h-1s.

### Estimation of gibberellic acid

The fine powder of the ground fruits at different developmental stages were placed into screw cap tubes filled with 30 ml methanol 70% (v/v) and kept overnight at 4°C. Supernatant was prepared by centrifugation and evaporation of methanol using a vacuum. The aqueous phase was partitioned with ethyl acetate after adjusting the pH at 8.5. The pH of the aqueous phase was again adjusted to 2.5 after removal of the ethyl acetate phase. The solution was partitioned with diethyl ether, and then passed through sodium sulfate. Diethyl ether was evaporated under vacuum, and dry residue containing GA_3_ was dissolved in 2.0 ml of absolute methanol. The GA_3_ analysis was performed using high performance liquid chromatography (HPLC) (Waters) equipped with reversed-phase column Crestpak C18 (150 mm × 4.6 mm i.d.; 5 µm) maintained at 30 ± 1°C. The mobile phase of acetonitrile-water (30:70%; v/v) was used with pH-4.5 and a flow rate of 1 ml/min. An injection volume of 10 µL was used for each analysis, and the wavelength of 208 nm was used for analysis.

### RNA isolation from the fruit at different developmental stages

RNA isolation was performed for each genotype at three developmental stages: on the day of anthesis (0 DAA), two days after anthesis (2 DAA) and four days after anthesis (4 DAA). Both pollinated and unpollinated ovaries from all three genotypes were collected for gene expression analysis. The flower buds were covered with a butter paper bag one day before anthesis and pollination on the next day morning with the male flowers from each genotype. Fresh pollen from the plants sprayed with silver thiosulphate to induce male flowers was used for pollinating the female buds of each genotype. After pollination, the buds were again covered with a butter paper bag to avoid any chance pollination. The fruit collected at different developmental stages was immediately immersed in liquid nitrogen and kept in −80°C until isolation of RNA. Tissues from three fruits at similar developmental stages were homogenised for RNA isolation. Total RNA was isolated using 100 mg of cucumber fruit from three genotypes at three different developmental stages using TRIZOL reagent.

### Primer designing

The Cucurbits Genomic Database (http://www.icugi.org/) was searched for important CK, AUX and GA biosynthesis genes by using the predicted amino acid sequences of *Arabidopsis* homologues as queries. Primers were made using the Primer3 Input Version 4.0 (https://primer3.ut.ee/) tool. The primers were synthesized for expression analysis (Integrated DNA Technologies, United States). The primer sequence of the important genes associated with biosynthesis of CK, AUX and GA are provided in the Supplementary Table S1.

### Phylogenetic study of the genes associated with cytokinin, auxin, and gibberellin biosynthesis

A total of 35 sequences of AUX, CK, and GA biosynthesis and degradation-related gene family of cucumber were extracted from the Cucurbits Genomic Database (http://www.icugi.org/). These were subjected to pairwise/multiple sequence alignment at default parameters for gap opening and gap extension penalty (i.e., 15 and 6.66, respectively in MEGA (Tamura et al., 2021)). The evolutionary history was inferred using the maximum likelihood method and the Tamura-Nei model (Tamura and Nei, 1993)]. An initial tree for the heuristic search was automatically obtained by applying the Neighbor-Join and BioNJ algorithms to a matrix of pairwise distances estimated using the Tamura-Nei model and then selecting the topology with the superior log likelihood value. The tree is drawn to scale, with branch lengths measured in the number of substitutions per site. A bootstrap method with 500 replications was employed at uniform rates among sites. The tree generated was scanned in Newick format to visualize and annotate in iTOL (Letunic and Bork, 2021).

### RT-PCR analysis for expression analysis of the important phytohormone biosynthesis and degradation genes

The cDNA was synthesized using a cDNA synthesis kit (GoScript™ Reverse Transcriptase) from 1 µg of isolated RNA. cDNA (200 ng) from all three genotypes of cucumber were used in a 10 μL Real-Time PCR machine (Bio-Rad, United States) using SYBR Green Master Mix (Promega, United States). Three biological replicates of cucumber fruit were taken to carry out qPCR reactions; a reference gene (Actin-mRNA) was used for the normalisation of gene expression. The relative level of gene expression was calculated by ΔCt method.

## Results

### Effects of exogenous application of auxin, cytokinin, and gibberellins in parthenocarpic fruit set

**FIGURE 1:**
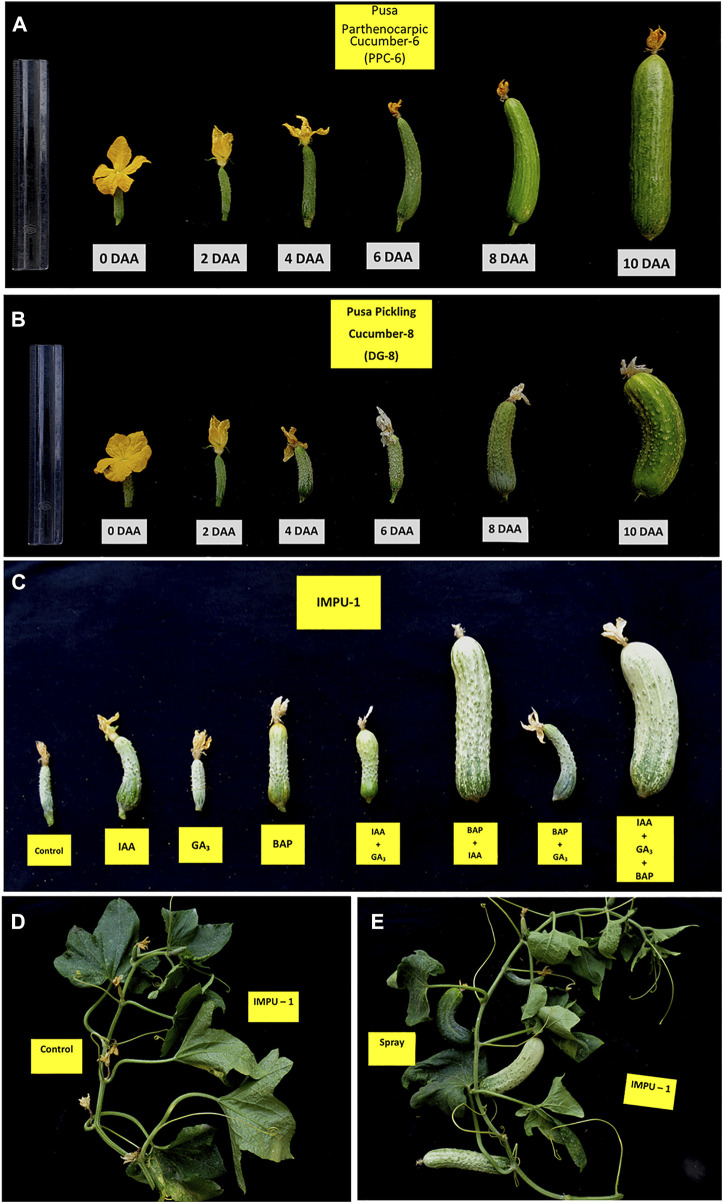
Parthenocarpic fruit development and the effects of the exogenous application of CK, AUX, and GA in the induction of parthenocarpy in cucumber. **(A)** Parthenocarpic fruit development in the slicing cucumber genotype PPC-6. **(B)** Parthenocarpic fruit development in the pickling cucumber genotype DG-8. **(C)** Effects of IAA, GA3, and Zeatin and their combination in fruit set and development in the non-parthenocarpic genotype IMPU-1. **(D)** Shrivelling of the flower buds of the non-parthenocarpic genotype, IMPU-1 under control with no pollination and exogenous application of phytohormones. **(E)** Normal fruit set and development in the genotype IMPU-1 with exogenous application of IAA + Zeatin in an unpollinated state.

It was found that the fruits of the parthenocarpic genotypes PPC-6 and DG-8 developed normally and attained a length of 15.5 cm and 8.9 cm, respectively at 10DAA, whereas more than 60% of the fruit of the unpollinated ovaries fell off the plant within 10 DAA in the genotype, IMPU-1. The retained fruits were shrivelled and did not develop beyond a certain length (Supplementary Figure S1). Fruit set in the non-parthenocarpic genotype IMPU-1 was observed through application of AUX, CK and GA and their combinations along with two parthenocarpic genotypes under control with no exogenous application. In the non-parthenocarpic genotype IMPU-1, the fruit set under control was very low (13.3%) and the initially retained fruits were shrivelled and fell down at a later stage. The highest parthenocarpic fruit set was recorded in the genotype PPC-6 (89.97%), followed by DG-8 (85.03%) under control with no exogenous application (Figures 1A,B). Exogenous application of the phytohormones had pronounced effects in the parthenocarpic development of fruit in the non-parthenocarpic genotype IMPU-1. The development of the fruit with the exogenous spray of CK, GA and AUX in the non-partheocarpic genotype IMPU-1 is presented in Figure 1C. The highest parthenocarpic fruit set (89.3%) was recorded with the combined application of IAA + Zeatin (Figures 1D,E) followed by Zeatin alone (80.8%) and IAA + Zeatin + GA_3_ (78.6%). Parthenocarpic fruit set in the non-parthenocarpic genotype with the exogenous application of IAA + Zeatin was on par with the parthenocarpic fruit set in the genotypes PPC-6 and DG-8 **(** Table 1)

**TABLE 1:** Effects of exogenous application of auxin, cytokinin, and gibberellins in the induction of parthenocarpic fruit set in cucumber.

Sl. no.	Genotype	Nature of the genotype	Treatments	Percentage fruit set	Development of fruit
1	Pusa Parthenocarpic Cucumber-6 (PPC-6)	Gynoecious Parthenocarpic	Control	89.97 a	Well developed
2	Pusa Pickling Cucumber-8 (DG-8)	Gynoecious Parthenocarpic	Control	85.03 ab	Well developed
3	IMPU-1 (F locus introgressed Pusa Uday)	Gynoecious non-parthenocarpic	Control	13.30 f	Shrivelled and fallen down at later stage
			IAA	67.77 d	Underdeveloped
			GA3	34.33 e	Underdeveloped
			BAP	80.80 bc	Developed
			IAA + GA3	68.97 d	Underdeveloped
			IAA + BAP	89.30 a	Well developed
			BAP + GA3	76.33 c	Developed
			IAA + GA3+BAP	78.60 c	Developed

Test of significance was done through Tukey’s honest significant difference (HSD) test at *p* = 0.05. The same letter in the column indicates no significant difference, and different letters indicate a significant difference among them.

### Estimation of endogenous AUX, CK, and GA in different developmental stages

**FIGURE 2:**
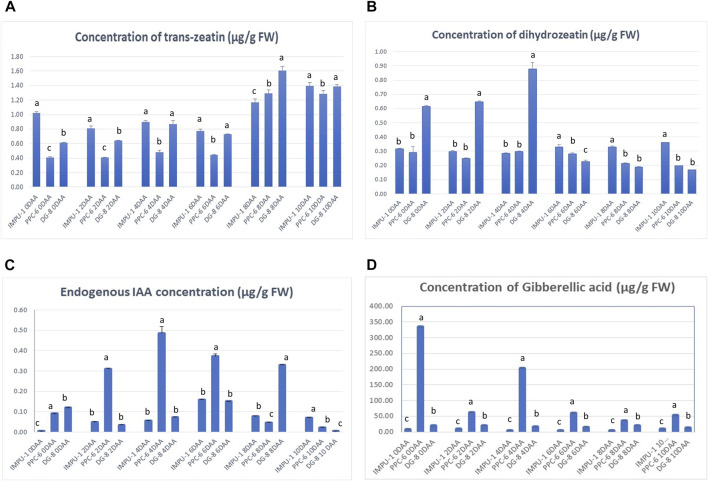
Endogenous concentration of **(A)** trans-zeatin, **(B)** dihydrozeatin **(C)** indole acetic acid (IAA) and **(D)** gibberellic acid (GA_3_) in the genotypes IMPU-1, PPC-6, and DG-8 at six different developmental stages in an unpollinated state. Test of significance performed through Tukey’s honest significant difference (HSD) test at *p* = 0.05. The same letter in the bar indicates no significant difference while different letters indicate significant difference among them.

The estimation of IAA and trans-zeatin and dihydrozeatin was undertaken in six development stages from the day of anthesis (0DAA) to ten days after anthesis (10DAA) in alternate days (Figure 2; Supplementary Table S2). The trans-zeatin concentration was initially higher in the non-parthenocarpic genotype IMPU-1. However, its concentration increased in the parthenocarpic genotypes with advancement in the developmental stages. At 8DAA, trans-zeatin was highest in the pickling-type cucumber genotype DG-8, followed by slicing-type PPC-6 (Figure 2A). In the genotype, DG-8 concentration of dihydrozeatin was highest in the initial stages to 4DAA and then declined. However, in the genotype PPC-6, its concentration was consistently lower across the developmental stages (Figure 2B). A significant difference in IAA concentration was recorded among the genotypes when comparing the parthenocarpic genotypes with the non-parthenocarpic genotype (Figure 2C). In addition, the concentration of IAA significantly varied among the slicing- and pickling-type parthenocarpic genotypes. On the day of anthesis, the highest concentration of IAA was recorded in the genotype DG-8, followed by PPC-6; it was lowest in the non-parthenocarpic genotype IMPU-1. While studying the concentration of IAA in the slicing cucumber genotype PPC-6, its concentration was recorded as increasing till 4DAA from the day of anthesis, and declined thereafter. In the pickling-type parthenocarpic genotype, the concentration of IAA was initially lower than PPC-6 but kept increasing till 8DAA before its decline. The IAA concentration was lower in the non-parthenocarpic genotype IMPU compared to the two parthenocarpic genotypes in all developmental stages. The concentration of GA_3_ varied significantly in different developmental stages among the genotypes studied (Figure 2D). The parthenocarpic genotype PPC-6 had the highest concentration of GA_3_ in all the developmental stages, whereas the non-parthenocarpic genotype IMPU-1 had the lowest. The concentration of GA_3_ in pickling cucumber genotype DG-8 was intermediate between PPC-6 and IMPU-1. In the parthenocarpic genotype PPC-6, concentration of GA_3_ was higher in the initial developmental stages and declined with the advancement of fruit development. In contrast, the processing parthenocarpic genotype DG-8 did not express such drastic changes in the endogenous GA_3_ level in different fruit developmental stages. The lowest concentration of GA_3_ was recorded in the non-parthenocarpic genotype IMPU-1 and was significantly lower than PPC-6 and DG-8 in all the six developmental stages (Figure 2D).

### Phylogenetic studies involving the CK, AUX, and GA biosynthesis-related genes

**FIGURE 3:**
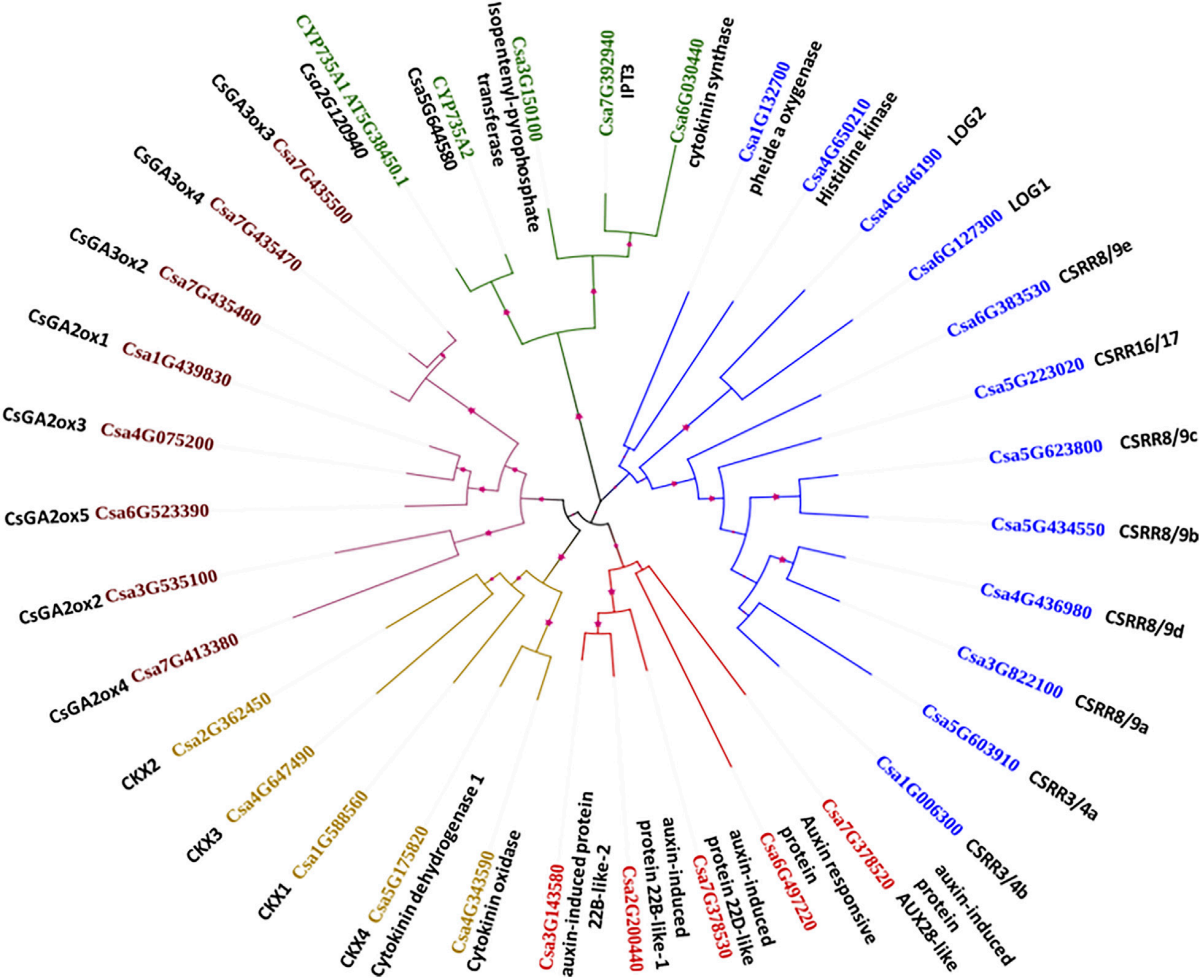
Phylogenetic analysis of the important cytokinin, auxin and gibberellin biosynthesis and degradation genes and their grouping into five major clusters.

Five major clusters were generated for the 35 CK, AUX and GA biosynthesis and degradation gene family sequences, as indicated by the different colours in Figure 3. The branch lengths are shown for each clade. Cluster I contains crucial genes for CK biosynthesis such as pheophorbide a oxygenase (Csa1G132700), CK signalling rich gene (*Csa4G650210*), *LOG1* (*Csa6G127300*), and *LOG2* (*Csa4G646190*), along with transcription factor-response regulators *CSRR8*/9e (*Csa6G383530*), *CSRR16*/17 (*Csa5G223020*), *CSRR8*/*9c* (*Csa5G623800*), *CSRR8*/*9b* (*Csa5G434550*), *CSRR8/9d* (*Csa4G436980*), *CSRR8/9a* (*Csa3G822100*), *CSRR3/4a* (*Csa5G603910*), and *CSRR3/4b* (*Csa1G006300*). Cluster II is represented by another group of CK biosynthesis genes such as cytokinin synthase (*Csa6G030440*), *IPT3* (*Csa7G392940*), *IPT* (*Csa3G150100*), *CYP735A1* (*Csa2G120940*), and *CYP735A2* (*Csa5G644580*). All the GA biosynthesis and degradation genes such as *CsGA3ox3* (*Csa7G435500*), *CsGA3ox4* (*Csa7G435470*), *CsGA3ox2* (*Csa7G435480*), *CsGA2ox1* (*Csa1G439830*), *CsGA2ox3* (*Csa4G075200*), *CsGA2ox5* (*Csa6G523390*), *CsGA2ox2* (*Csa3G535100*), and *CsGA2ox4* (*Csa7G413380*) were grouped together in Cluster III. Cluster IV consists of CK degradation genes *CKX2* (*Csa2G362450*), *CKX3* (*Csa4G647490*), *CKX1* (*Csa1G588560*), CKX4/cytokinin dehydrogenase 1 (*Csa5G175820*), and cytokinin oxidase (*Csa4G343590*); cluster V contained auxin-induced protein 22B-like-2 (*Csa3G143580*), auxin-induced protein 22B-like-1 (*Csa2G200440*), auxin-induced protein 22D-like (*Csa7G378530*), auxin-induced protein AUX28-like (*Csa7G378520*), and auxin-responsive protein (*Csa6G497220*).

### Relative expression of genes associated with CK biosynthesis

**FIGURE 4:**
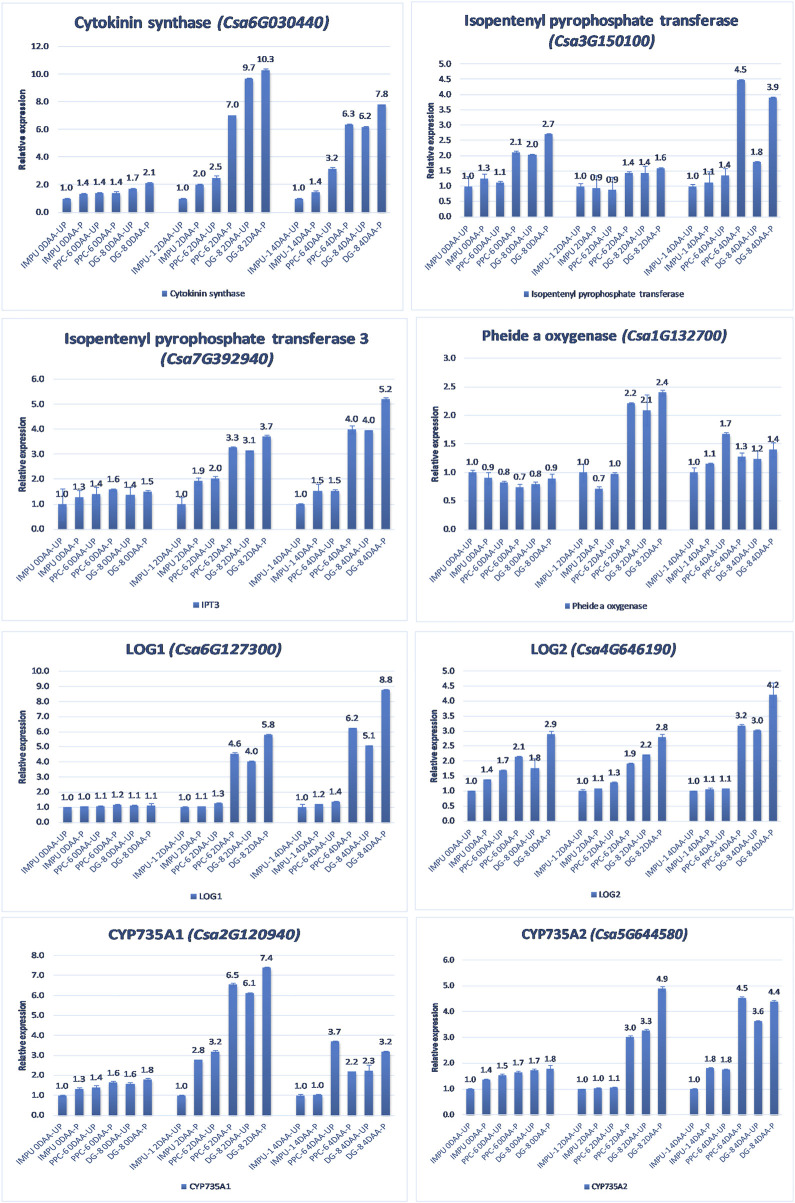
Relative expression of the cytokinin biosynthesis genes in the genotypes IMPU-1, PPC-6, and DG-8 under three different developmental stages of pollinated and unpollinated ovaries.

**FIGURE 5:**
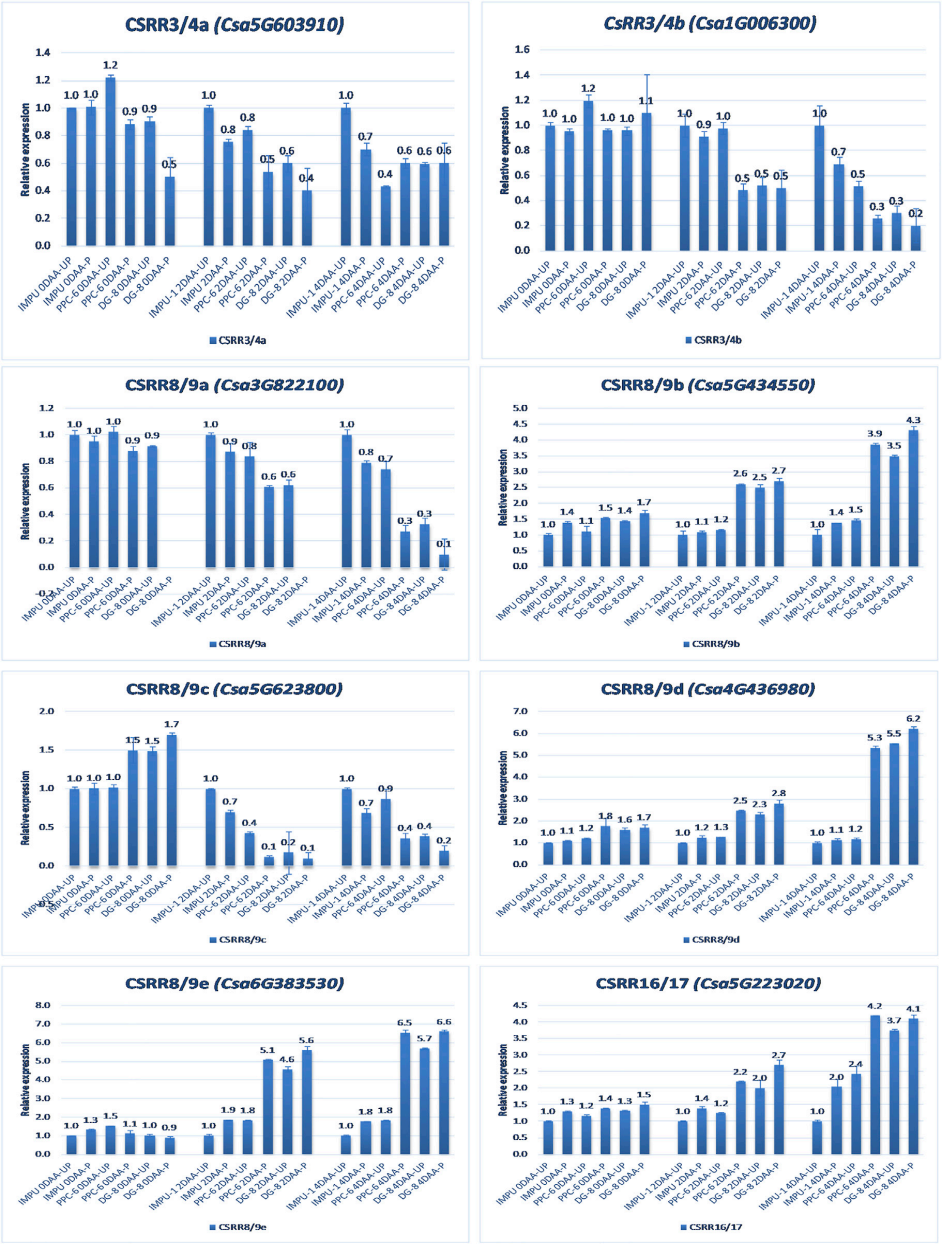
Relative expression of the cytokinin transcription factor-response regulators (RRs) in the genotypes IMPU-1, PPC-6, and DG-8 under three different developmental stages of pollinated and unpollinated ovaries.

**FIGURE 6:**
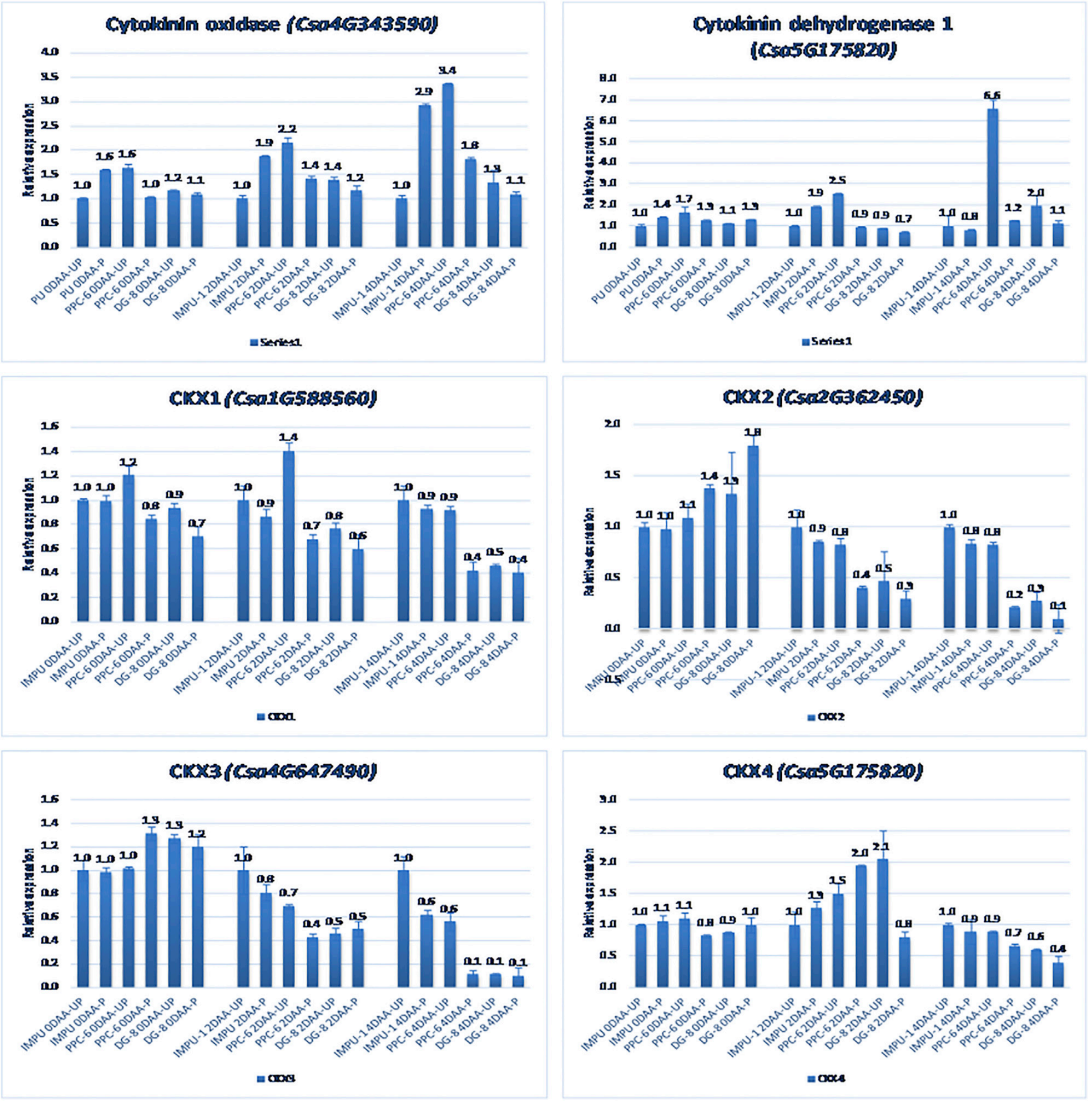
Relative expression of the cytokinin degradation genes in the genotypes IMPU-1, PPC-6, and DG-8 under three different developmental stages of pollinated and unpollinated ovaries.

A total of 22 CK biosynthesis and degradation-related genes and transcripts were investigated at three different developmental stages using both pollinated and unpollinated flower buds. The relative expression of the parthenocarpic genotypes were calculated in comparison with the non-parthenocarpic genotype IMPU-1. Cytokinin synthase was significantly up-regulated in the parthenocarpic genotypes PPC-6 and DG-8, both pollinated and unpollinated. At 2DAA, the relative expression was higher in both parthenocarpic genotypes; its expression later declined. It was also significant that a higher expression of cytokinin synthase was recorded at a pollinated state compared to the unpollinated state in both the parthenocarpic and non-parthenocarpic genotypes. While studying the relative expression of *IPT*, its expression was found to be higher in the initial stage (0DAA) and then declined at 2DAA. However, the expression of *IPT* again peaked at 4DAA, particularly in the pollinated ovaries of the parthenocarpic genotypes. Similarly, consistent up-regulation of *IPT3* was recorded from the anthesis to 4DAA in both the pollinated and unpollinated ovaries of the genotypes PPC-6 and DG-8. The expression of cytokinin synthase, *IPT* and *IPT3* were higher in the pollinated flower buds of the non-parthenocarpic genotype IMPU-1 than in the unpollinated ovaries. Up-regulation of the *PaO, LOG1*, and *LOG2* were observed in the parthenocarpic genotypes PPC-6 and DG-8 in both pollinated and unpollinated states. The extent of up-regulation was higher in the case of *LOG1* from 2DAA onwards (Figure 4). Eight CK response regulator genes were studied for their relative expression in parthenocarpic and non-parthenocarpic genotypes (Figure 5). Among them, *CsRR8/9b, CsRR8/9d*, and *CsRR8*/9e were significantly up-regulated in the parthenocarpic genotypes and *CSRR8*/*9a*, *CSRR3/4a*, and *CSRR3/4b* were down-regulated in the parthenocarpic genotypes, with the development of the ovaries from anthesis onwards. The relative expression of the cytokinin oxidase/dehydrogenase (*CKXs*) was also investigated in the three genotypes in both pollinated and unpollinated states. It was found that *CKX2 and CKX3* were down-regulated in the parthenocarpic genotypes at a later stage after initial up-regulation. Cytokinin oxidase and cytokinin dehydrogenase were up-regulated in the parthenocarpic genotypes: the extent of the up-regulation was stronger in the genotype PPC-6 than DG-8. In the genotypes PPC-6 and DG-8, stronger expressions of *CKX1 and CKX4*, respectively, were recorded at 2DAA, before declining thereafter. However, no significant difference in the expression of *CKXs* was recorded in the pollinated and unpollinated flowers of the non-parthenocarpic genotype IMPU-1 (Figure 6).

### Relative expression of genes associated with AUX and GA biosynthesis and degradation

**FIGURE 7:**
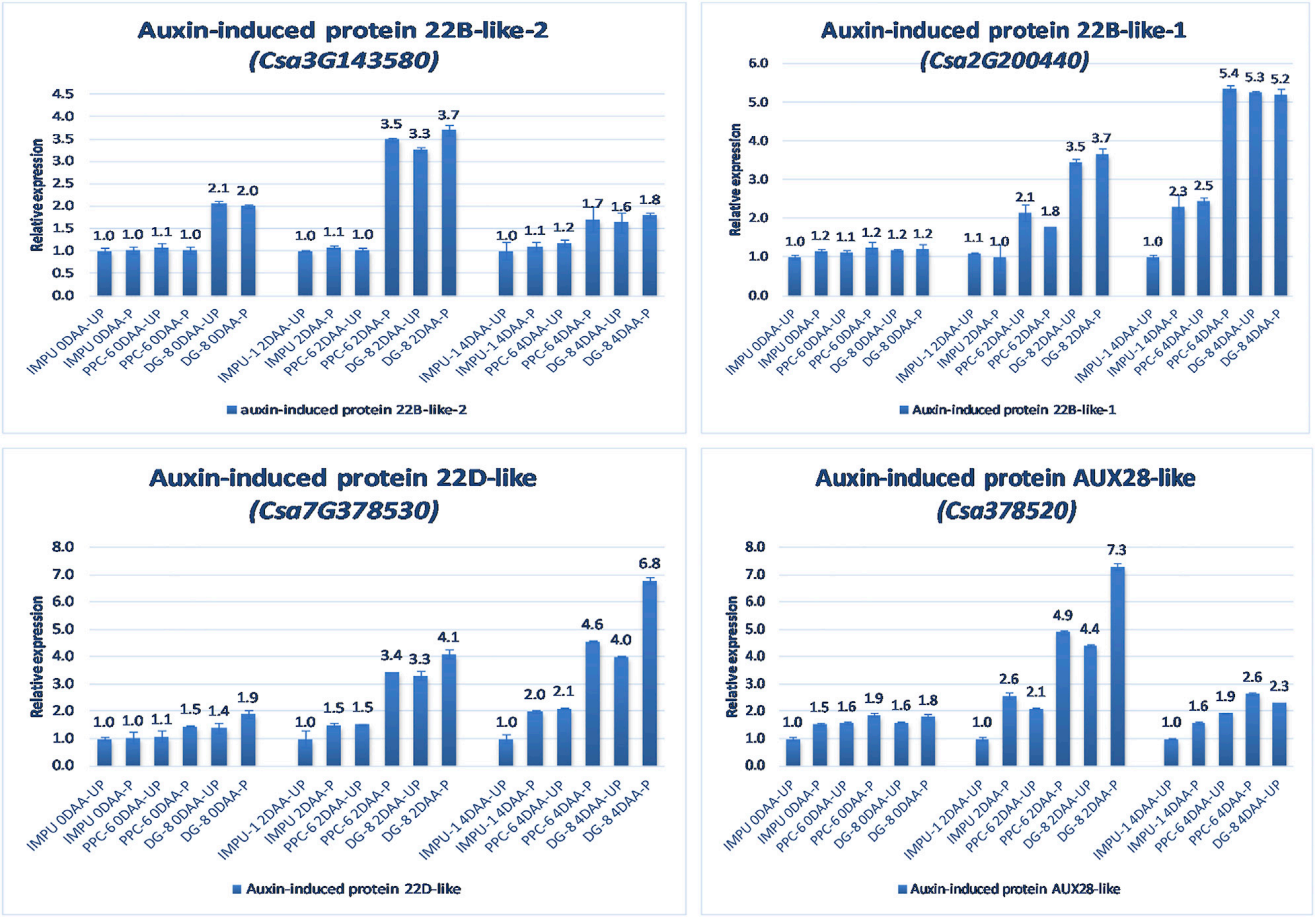
Relative expression of the important auxin-induced proteins in the genotypes IMPU-1, PPC-6, and DG-8 under three different developmental stages of pollinated and unpollinated ovaries.

**FIGURE 8:**
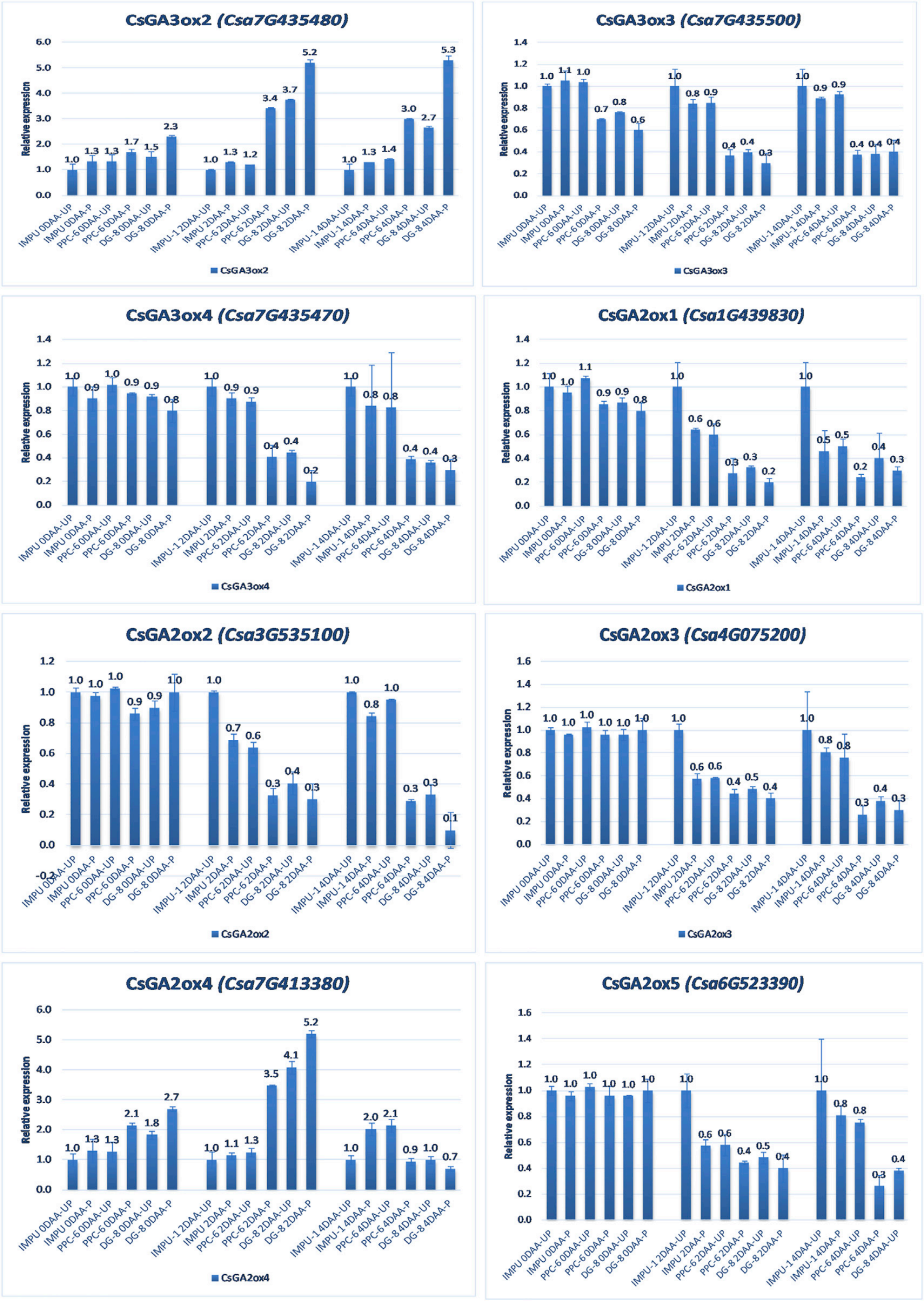
Relative expression of the important gibberellin biosynthesis and degradation genes in the genotypes IMPU-1, PPC-6, and DG-8 under three different developmental stages of pollinated and unpollinated ovaries.

The relative expression of the four AUX-induced proteins and eight gibberellin oxidases were also investigated in the three sets of genotypes at different time intervals. Among the AUX induced proteins, Auxin-induced protein 22B-like-2 and Auxin-induced protein AUX28-like were significantly up-regulated in the parthenocarpic genotypes at 2DAA. However, Auxin-induced protein 22D-like and Auxin-induced protein 22B-like-2 had significantly higher expression from the day of the anthesis to 4DAA (Figure 7). It was interesting that the level of expression of the auxin-induced proteins in the slicing-type parthenocarpic genotype PPC-6 was relatively lower in the unpollinated state than the pollinated state in the parthenocarpic pickling-type genotype DG-8. Among the eight gibberellin biosynthesis pathway-related genes taken for the study, six were either down-regulated or did not differ significantly from the non-parthenocarpic genotype IMPU-1 under both pollinated and unpollinated states. However, the expression of *CsGA3ox2* was higher in the parthenocarpic genotypes in all the developmental stages; *CsGA2ox4* was up-regulated in the initial development stages at 0DAA and 2DAA and was later down-regulated at 4DAA (Figure 8). There was no significant difference in the pollinated and unpollinated ovaries of the non-parthenocarpic genotype IMPU-1 at different stages for pollinated and unpollinated ovaries for both AUX and GA biosynthesis-related genes.

## Discussion

Successful completion of pollination and a unique double fertilization is the prerequisite for initial fruit setting and further development of fruits in most of the angiosperms (Raghavan, 2003). However, a few selected organisms can produce fruit without requiring pollination and fertilization—termed ‘parthenocarpy’’. Cucumber is one such model organism with abundant germplasm which has the typical phenomenon of parthenocarpy. In case of non-parthenocapic genotypes, successful pollination and fertilization results in formation of seed which promotes synchronized cell division and fruit growth. A number of studies have indicated the role of three important phytohormones—AUX, GAs, and CKs—in the regulation of fruit set. These three hormones are required in combinations for fruit set and further development because individually they can only set fruit to a certain extent and cannot support the development of fully-grown fruit **(** Mariotti et al., 2011; Sharif et al., 2022). Cross-talk between the phytohormones is well established in the regulation of different biological processes in plants (Coenen and Lomax 1997). Induction of parthenocarpy in various plant species is mainly regulated by AUX, GAs, and CKs (Fu et al., 2008; Serrani et al., 2008; Sharif et al., 2022).

The non-partheocapic gynoecious genotype IMPU-1 did not set fruits parthenocarpically, although a few ovaries were enlarged in the initial stage and then shrivelled and fell off later. However, the parthenocarpic lines PPC-6 and DG-8 successfully developed fruit without pollination and expressed a very high degree of parthenocarpy. Exogenous application of AUX, GA, and CK individually and in combination had a very significant role in fruit set and development in the non-parthenocarpic genotype. The highest percentage of fruit set and the normal development of fruit were observed when AUX and CK were applied in combination. Individual application of CK was also effective in fruit set and their enlargement. However, individual application of AUX and GA were less effective as their application enabled fruit set but could not carry the fruit to full development. A better suitability of CK and AUX in the development of parthenocarpic fruit in cucumber has been demonstrated by Su et al. (2021). The exogenous application of growth hormones like CKs and GAs were found to be effective in developing parthenocarpic fruits in non-parthenocarpic genotypes of tomato (Matsuo et al., 2012), eggplant (Donzella et al., 2000), and pear (Niu et al., 2014).

Varied concentrations of trans-zeatin and dihydrozeatin were recorded in different developmental stages; at the initial stage, a higher concentration of trans-zeatin was recorded in the genotype IMPU-1 in an unpollinated state. In contrast, dihydrozeatin was present in higher concentrations in the genotype DG-8 in the initial stages, indicating a different role for the CKs in regulating the parthenocarpy of slicing and pickling cucumber. A lower concentration of *trans*-zeatin in the parthenocarpic genotypes in initial stages of development might be due to a stronger expression of some CK degradation enzymes such as cytokinin oxidase, *CKX2*, and *CKX4* in the initial stages. The expression level of the CK biosynthesis and degradation genes were different even among the parthenocarpic lines. The endogenous concentration of the CKs was ultimately determined by the homeostasis between CK biosynthesis and catalytic genes and regulators (Frébort et al., 2011). The relatively weaker expression of the *CKXs* in the pollinated ovules was due to the negative regulation of these enzymes when there was successful fertilization and seed development. However, the concentration of AUX was higher in the unpollinated ovaries of the parthenocarpic genotypes at all the developmental stages. However, the concentration of IAA was highest in the slicing-type genotype at 4DAA and then started to decline. On the other hand, it increased in the pickling-type genotype to 8DAA before declining. This trend indicated potentially different genetic mechanisms that control parthenocarpy in slicing- and pickling-type cucumbers. Different genomic locations determining parthenocarpy in these types of cucumber were reported by Wu et al. (2015), Lietzow et al. (2016) and Niu et al. (2020). Higher concentrations of AUX in the ovules of the parthenocarpic cucumber genotypes were earlier reported by Su et al. (2021) and Qian et al. (2018).

CKs are important regulators for the development of fruit. CK-regulated cell division and development of fruit have been reported in tomato (Matsuo et al., 2012). The findings of the present study also revealed a higher concentration of CK in the ovaries of the parthenocarpic genotypes PPC-6 and DG-8, thus establishing the role of CK in parthenocarpic fruit development in cucumber genotypes. A higher expression of the genes cytokinin synthase, isopentenyl pyrophosphate transferase 3, and *LOG2* was recorded in the parthenocarpic genotypes in different developmental stages. It was also notable that the expression level of these important genes associated with cytokinin biosynthesis was higher in the pollinated flower buds than in unpollinated ones. In the non-parthenocarpic genotype IMPU-1, higher expression of the CK synthase and *IPT3* was recorded in the pollinated flower buds. Homeostasis between CK synthesis and catabolism determined spatial and temporal biosynthesis in different parts of the plant (Frébort et al., 2011). The production of isopentenyladenine nucleotides catalysed by adenosine phosphate-isopentenyl transferase (*IPT*) involves the first step of CK biosynthesis (Sakakibara, 2005; Sakakibara, 2005). The hydroxylation of the prenyl side-chain of isopentenyl adenosine phosphates is mediated by a cytochrome P450 mono-oxygenase (*CYP735A*) to produce trans-zeatin-type species. The nucleotide precursors are converted into their active forms by the *LONELY GUY (LOG)* while degradation of CK is catalysed by CK oxidases (*CKXs*) (Sakakibara, 2005; Sakakibara, 2006; Schäfer et al., 2015). Up-regulation of the genes associated with CK biosynthesis such as *CYP735A1*, *CYP735A2*, and *LOG1* and down-regulation of the CK dehydrogenase genes *CKX1* and *CKX3* was reported to be the reason behind parthenocarpic fruit development in cucumber (Su et al., 2021). Our study also revealed the enhanced expression of the CK biosynthesis genes such as *IPT*, *IPT1*, *IPT3*, *LOG1*, *LOG2*, *CYP735A1,* and *CYP735A2* and reduced expression of *CKX1, CKX2,* and *CKX3* in the parthenocarpic genotypes PPC-6 and DG-8. Therefore, the role of the CK in parthenocarpic fruit set of both slicing and pickling cucumber is established. However, the extent of up-regulation and down-regulation of CK biosynthesis genes varied among the slicing and pickling parthenocarpic genotypes. In addition, a higher expression of the CK biosynthesis-related genes was recorded in the pollinated ovules compared to the unpollinated buds. Enhanced expression of CK biosynthesis genes (*SlIPT3*, *SlIPT4*, *SlLOG6*, and *SlLOG8*) was associated with CPPU-induced parthenocarpy in tomato (Matsuo et al., 2012). Transcription factor-response regulators (RRs) are vital for the interaction of CK with an array of other hormones via the multistep phosphorelay system (MSP) (Arkhipov et al., 2019). CSRR8/9a was down-regulated in the parthenocarpic genotypes with the advancement of developmental stages in both unpollinated and pollinated states. Type-A RR genes are classified as negative feedback regulators of CK signalling (Osugi and Sakakibara. 2015; Kieber and Schaller, 2018). CK signalling genes such as *CsRR3/4a*, *CsRR3/4b, CsRR8/9a*, and *CsRR8*/9c are reported to be strongly expressed in the non-parthenocarpic cucumber genotype, thus elucidating their negative regulation in parthenocarpic fruit development (Su et al., 2021). The present study also revealed the negative feedback of the *CsRR3/4a, CsRR3/4b, CsRR8/9a,* and *CsRR8*/9c genes in parthenocarpic fruit development. However, positive regulation of the *CSRR8/9b, CSRR8/9d, CSRR8/9e,* and CSRR16/17 genes were also revealed, which was not known earlier in the parthenocarpic fruit development of cucumber. The role of the CK signal transduction in the induction of parthenocarpy in cucumber is established from the finding of the study. Significant up-regulation of the *IPTs* in the parthenocarpic genotypes explained CK regulated parthenocarpy in cucumber. The results of this study indicated the role of Ck biosynthesis genes in parthenocarpic fruit set through their up-regulation in the parthenocarpic genotypes. However, the AUX and GA metabolisms were also found to be intermingled with CK pathways in determining parthenocarpy.

AUX signalling genes are responsible for the dynamic role of AUX-regulated growth activities (Sharif et al., 2022). The roles of AUX signal transduction genes in parthenocarpic fruit formation have been more intensively studied than the AUX biosynthesis and transportation genes. Auxin-induced protein 22B-like-1, auxin-induced protein 22B-like-2, auxin-induced protein 22D-like, and auxin-induced protein AUX28-like were strongly expressed in the parthenocarpic genotypes at different developmental and pollinated stages of the ovaries. However, their relative expression varied among the slicing and pickling parthenocarpic cucumber genotypes. Their expression was also enhanced in the pollinated state in the non-parthenocarpic genotype IMPU-1 and slicing cucumber genotype PPC-6. Su et al. (2021) also reported the positive expression of AUX signal transduction genes such as *AUX 22A-like-1*, *AUX22B-like-2,* and *AUX 28-like* in the parthenocarpic cucumber genotype DDX. Auxin is well known for its role in the development of fleshy fruits and reported to be integral to the initial signal for fertilisation and increased fruit size through its influence in cell division and expansion (Godoy et al., 2021). Once the fruits set parthenocarpically, their further development is influenced by auxins, evidenced by up-regulation of the auxin biosynthesis-related genes in the later stages of fruit development in the parthenocarpic genotypes.

Gibberellin is another crucial hormone for fruit set and development (Wang et al., 2020). GA13-oxidase (*GA13ox*), GA 20-oxidase (*GA20ox*), and GA 3-oxidase (*GA3ox*) enzymes are involved in the biosynthesis of GA_1_ and GA_4_ (Hedden, 2020). The primary GA deactivation enzyme GA2-oxidase determines the concentrations of these active GAs (Hedden, 2020). On the other hand, the degradation of GA_1+4_ (active GA) is catalysed by a major GA degradation gene, *GA2ox*, which is crucial for GA homeostasis in plants (Martínez-Bello et al., 2015). The role of the GA biosynthesis and degradation genes in relation to parthenocarpic fruit development in cucumber is unknown. In our study, *CsGA3ox2* was strongly expressed in the parthenocarpic genotypes PPC-6 and DG-8 whereas *CsGA3ox4* was strongly expressed in the parthenocarpic genotypes at the initial stage of ovary development (2DAA); its expression later declined, indicating the stage-specific role of this enzyme in induction of parthenocarpy. The enzymes *CsGA3ox3*, *CsGA3ox4, CsGA2ox1, CsGA2ox2, CsGA2ox3,* and *CsGA2ox5* were weakly expressed in the parthenocarpic genotypes, the pollinated state indicating their negative regulation in the induction of parthenocarpic fruit in cucumber. In pear, the dominant expression of the GA biosynthesis gene *GA20ox* was reported in the pollinated fruit (Wang et al., 2020). Increased GA_4_ levels in tomato and pear through overexpression of *SlGA20ox* and *PbGA20ox* genes, respectively, resulted in parthenocarpic fruit development (Wang et al., 2020). In tomato, fruit setting was associated with the up-regulation of GA biosynthesis genes (*SlGA20ox1*, *SlGA20ox2*, and *SlGA20ox3*) and the down-regulation of the GA deactivation gene *SlGA2ox1* (Okabe et al., 2019). Varied concentrations of endogenous CKs and AUX and their differential expression at different developmental stages established the potentially different molecular mechanisms and regulatory networks that determine parthenocarpy in slicing and pickling cucumbers. The findings of the present study reveal the cross-talk between the important plant hormones in relation to parthenocarpic fruit development in cucumber.

## Data Availability

The original contributions presented in the study are included in the article/Supplementary Material; further inquiries can be directed to the corresponding authors.

## Supplementary material

SUPPLEMENTARY TABLE S1 List of the genes, respective gene IDs and primer sequence used for RT-PCR analysis.

SUPPLEMENTARY TABLE S2 Concentration of the different phytohormones at six developmental stages in three cucumber genotypes.

SUPPLEMENTARY FIGURE S1 Sequential development of the unpollinated fruits in the genotypes IMPU-1, PPC-6 and DG-8 at different developmental stages.
