# Supplementary figures and images for: Cross-talk between the cytokinin, auxin, and gibberellin regulatory networks in determining parthenocarpy in cucumber
Source: Front Genet. 2022 Aug 26;13:957360. doi: 10.3389/fgene.2022.957360 (PMC9459115; doi:10.3389/fgene.2022.957360)

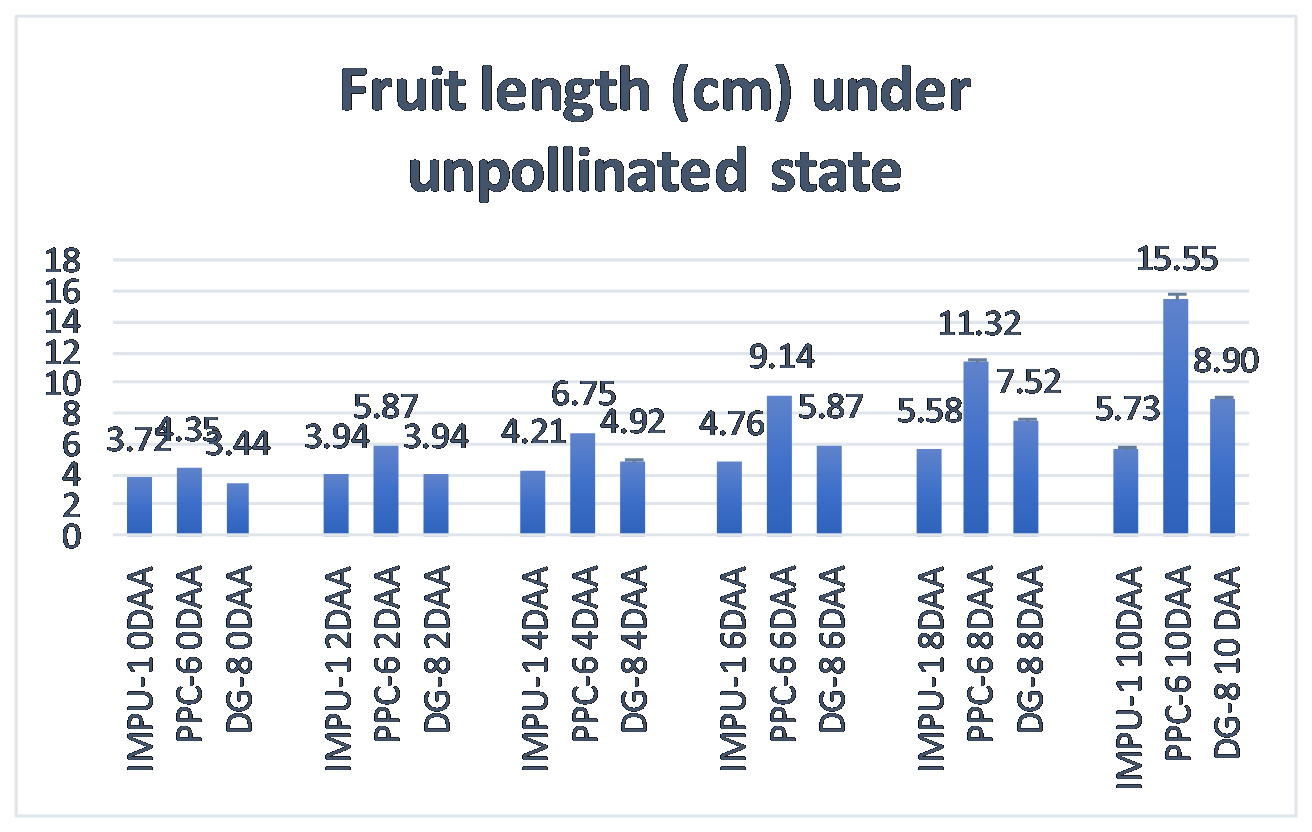

Supplement: Supplementary file 1 [file Image1.TIF]
