# Supplementary material for: Cross-talk between the cytokinin, auxin, and gibberellin regulatory networks in determining parthenocarpy in cucumber
Source: Front Genet. 2022 Aug 26;13:957360. doi: 10.3389/fgene.2022.957360 (PMC9459115; doi:10.3389/fgene.2022.957360)
Supplement: Supplementary file 3 [file DataSheet1.docx]

**Supplementary Table 2.** Concentration of the different phytohormones at 6 developmental stages in three cucumber genotypes

**Indole Acetic Acid:**

| **Genotype** | **0DAA** | **2DAA** | **4DAA** | **6DAA** | **8DAA** | **10DAA** |
| --- | --- | --- | --- | --- | --- | --- |
| *Indole Acetic Acid (ppm)* | | | | | | |
| IMPU-1 | 0.005 c | 0.049 b | 0.055 b | **0.158 b** | 0.078 b | 0.071 a |
| Pusa Parthenocarpic cucumber-6 | 0.091 a | 0.310 a | **0.485 a** | **0.372 a** | 0.045 c | 0.023 b |
| Pusa Pickling Cucumber-8 | 0.019 b | 0.035 b | 0.072 b | **0.151 b** | **0.330 a** | 0.006 c |

Test of significance was done through Tukeys's Honest Significant Difference (HSD) Test at p=0.05. The same letter in the column indicated no significant difference and different letters indicated significant difference among them.

**Trans Zeatin:**

| **Genotype** | **0DAA** | **2DAA** | **4DAA** | **6DAA** | **8DAA** | **10DAA** |
| --- | --- | --- | --- | --- | --- | --- |
| *Trans-zeatin* | | | | | | |
| IMPU-1 | 1.02 a | 0.81 a | 0.90 a | 0.77 a | 1.17 c | 1.39 a |
| Pusa Parthenocarpic cucumber-6 | 0.41 c | 0.41 c | 0.48 b | 0.44 b | 1.29 b | 1.29 b |
| Pusa Pickling Cucumber-8 | 0.61 b | 0.64 b | 0.87 a | 0.73 a | 1.61 a | 1.39 a |

Test of significance was done through Tukeys's Honest Significant Difference (HSD) Test at p=0.05. The same letter in the column indicated no significant difference and different letters indicated significant difference among them.

**Dihydrozeatin**

| **Genotype** | **0DAA** | **2DAA** | **4DAA** | **6DAA** | **8DAA** | **10DAA** |
| --- | --- | --- | --- | --- | --- | --- |
| *Dihydrozeatin* | | | | | | |
| IMPU-1 | 0.32 b | 0.30 b | 0.28 b | 0.33 a | 0.33 a | 0.36 a |
| Pusa Parthenocarpic cucumber-6 | 0.29 b | 0.25 b | 0.30 b | 0.28 b | 0.21 b | 0.20 b |
| Pusa Pickling Cucumber-8 | 0.61 a | 0.65 a | 0.88 a | 0.23 c | 0.19 b | 0.17 b |

Test of significance was done through Tukeys's Honest Significant Difference (HSD) Test at p=0.05. The same letter in the column indicated no significant difference and different letters indicated significant difference among them.

**Gibberellic Acid (GA_3_)**

| **Genotype** | **0DAA** | **2DAA** | **4DAA** | **6DAA** | **8DAA** | **10DAA** |
| --- | --- | --- | --- | --- | --- | --- |
| *Gibberellic acid* | | | | | | |
| IMPU-1 | 7.97 c | 9.57 c | 4.42 c | 3.09 c | 3.31 c | 8.47 c |
| Pusa Parthenocarpic cucumber-6 | 333.37 a | 61.99 a | 201.51 a | 59.60 a | 35.29 a | 51.73 a |
| Pusa Pickling Cucumber-8 | 18.85 b | 19.18 b | 15.55 b | 13.87 b | 19.09 b | 12.94 b |

Test of significance was done through Tukeys's Honest Significant Difference (HSD) Test at p=0.05. The same letter in the column indicated no significant difference and different letters indicated significant difference among them.
